# Multiple independent sampling within medical school admission interviewing: an “intermediate approach”

**DOI:** 10.1007/s40037-016-0298-9

**Published:** 2016-09-16

**Authors:** Mark D. Hanson, Nicole N. Woods, Maria Athina Martimianakis, Raj Rasasingham, Kulamakan Kulasegaram

**Affiliations:** 1 Department of Psychiatry, Faculty of Medicine, University of Toronto, Toronto, Ontario Canada; 2 The Wilson Centre, University of Toronto, Toronto, Ontario Canada; 3 Hospital for Sick Children, Toronto, Ontario Canada; 4 Humber River Regional Hospital, Toronto, Ontario Canada

**Keywords:** Admissions, Interviewing, Competencies, Caring, Empathy

## Abstract

**Introduction:**

Balancing reliability and resource limitations as well as recruitment activities during admission interviews is a challenge for many medical schools. The Modified Personal Interview (MPI) has been shown to have good psychometric properties while being resource efficient for specialized admission interviews. We describe implementation of an MPI adaptation integrating psychometric rigour alongside resourcing and recruitment goals for larger-scale medical school admission interviewing at the University of Toronto.

**Methods:**

The MPI was implemented during the 2013–2014 admission cycle. The MPI uses multiple independent sampling by having applicants interviewed in a circuit of four brief semi-structured interviews. Recruitment is reflected in a longer MPI interviewing time to foster a ‘human touch’. Psychometric evaluation includes generalizability studies to examine inter-interview reliability and other major sources of error variance. We evaluated MPI impact upon applicant recruitment yield and resourcing.

**Results:**

MPI reliability is 0.56. MPI implementation maintained recruitment compared with previous year. MPI implementation required 160 interviewers for 600 applicants whereas for pre-MPI implementation 290 interviewers were required to interview 587 applicants. MPI score correlated with first year OSCE performance at 0.30 (*p* < 0.05).

**Discussion:**

MPI reliability is measured at 0.56 alongside enhanced resource utilization and maintenance of recruitment yield. This ‘intermediate approach’ may enable broader institutional uptake of integrated multiple independent sampling-based admission interviewing within institution-specific resourcing and recruitment goals.

## What this paper adds

- Medical schools commonly use personal interviews in admissions; yet psychometric studies show them to have low reliability and little predictive validity. One solution is multiple independent sampling with the Multiple Mini-Interview (MMI) being the exemplar. However, MMIs have substantial resourcing demands and potential negative recruitment impacts. The Modified Personal Interview (MPI) seeks to address these two issues with fewer interview stations [4] but a longer interview duration. In this paper we show its measurement utility for general MD admissions. The MPI can be an alternative for medical schools seeking to enhance measurement properties of interviews within resource and recruitment constraints.

## Introduction

Admission to medical school is predicated upon applicants possessing academic ability alongside a range of key personal competencies [1]. Admission personal interviews are commonly used to rate personal competencies. However, evaluation of the admission personal interview demonstrated low inter-rater reliability and prediction of performance. Albanese et al. [2] reported that panels with multiple interviewers jointly interviewing applicants appeared to have minimal impact upon inter-rater reliability. An Association of American Medical Colleges (AAMC) 2011 Analysis in Brief reported that the admission personal interview has been slow to change with it still being the default interview format [3]. Koenig et al. reported that schools used interview formats ranging from unstructured to structured with the most commonly used format being semi-structured [1]. This publication [1] noted interviewing formats warranting attention, such as the Multiple Mini-Interview (MMI) [4]. Despite low measurement properties, admission personal interviews provided institutional opportunities to present a ‘human touch’ reflective of the importance ascribed to person-to-person interactions and personal attributes in the selection of future physicians within an otherwise stressful application process [2]. Moreover, a recent AAMC Last Page publication identified admission interviews as important to applicants’ decision-making regarding medical school selection [5]. Substantial recruitment value was ascribed to interviews enabling interaction with senior medical students, faculty and administrative staff.

One solution to optimize interview reliability in admission interviews is adoption of multiple independent sampling (MIS). Instead of one evaluation of an applicant, several, independent evaluations of the same applicant are averaged to generate an applicant’s evaluation [6]. However, uptake by American medical schools of the MMI, a MIS-based interview format, has been limited due to 1) its substantial resource requirements and 2) associated modifications of campus recruitment activities and diminution of recruitment advantages associated with admission personal interviews [6]. Of note, successful applicant recruitment contributes to student selectivity within a medical school’s admission processes and its associated prestige ranking [7], with prestige being a vaunted element of today’s medical school environment [7–10]. Furthermore, recruitment advantage for admission personal interviews above the MMI has also been reported for Emergency Residency selection decision-making [11]. To address MIS and the combined issues of reliability, recruitment and resourcing within admission decision-making, Axelson and Kreiter investigated adapting MIS to admission personal interviewing. For their study, they conducted secondary data analysis of multiple admission interview cohorts; predicting reliability of 0.48 for three brief (one independent interviewer) personal interviews to 0.73 for nine brief (one independent interviewer) personal interviews. They recommended adapting admission personal interviewing via MIS as an ‘intermediate approach’ for schools wishing to address this combination of issues yet unable or unwilling to adopt MIS as utilized in the MMI, given its resourcing and/or recruitment impacts [6].

**Fig. 1:**
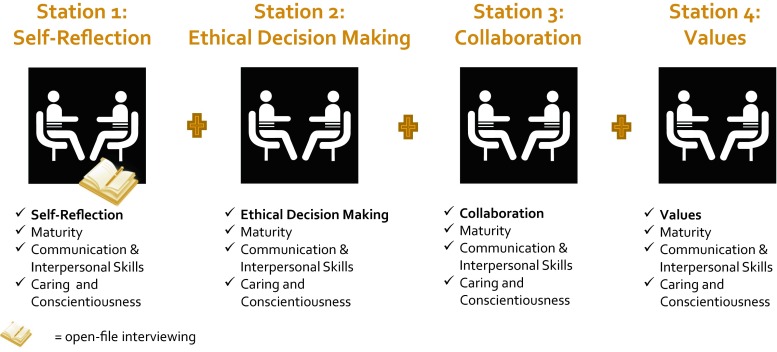
Modified Personal Interview (MPI)

Inspired by the ‘intermediate approach’ model of Axelson and Kreiter [6] we developed the Modified Personal Interview (MPI) employing MIS for small-scale specialized medical student selection tasks (leadership [10] and MD PhD [12]). As a medical school intent upon bringing MIS to large-scale admission interviewing but unwilling to implement the MMI due to recruitment and resourcing issues we adapted our MPI small-scale model to our large-scale admission context. (Please see Appendix A and Fig. 1 for a description of this large-scale MPI adaptation). Cognizant of the aforementioned combination of admission issues, we evaluated outcomes relevant to each issue through our MPI implementation large-scale process: MPI reliability, MPI predictive validity plus MPI admission recruitment and resourcing outcomes. We hypothesized 1) MPI reliability to be intermediate between 0.48 to 0.73 as predicted by Axelson and Kreiter [6], 2) positive MPI predictive validity for in-programme skills assessments such as a first year objective structured clinical examination (OSCE) and 3) maintenance of recruitment outcomes and resource efficiency.

## Methods

The study’s methods were selected to evaluate the MPI against multiple admission outcomes; 1) psychometric evaluation of MPI reliability and predictive validity and 2) institutional recruitment and resourcing outcomes associated with MPI implementation.

### MPI reliability

#### Analyses

We examined the reliability of 2013/2014 implementation administration of the MPI. Analysis of reliability used generalizability theory via G‑string IV (perd.mcmaster.ca). Generalizability theory allows modelling of multiple sources of variance and the impact of changing the number of measurements via decision studies (D-studies). Our generalizability model examined the error due to stations and items nested in each station. The purpose was to evaluate inter-interview reliability across four interviews and to determine the amount of error due to rater, station type, and items evaluated within each interview.

To evaluate whether interview and interviewer type were influencing scoring, we conducted two analyses. Firstly, we conducted item-total correlations for all of the station types as well as compared differences in scoring using a factorial repeated measures ANOVA with station type as the repeated measures variable and circuit as the between-subjects variable. Secondly we conducted a repeated measures ANOVA between rater types (medical students, residents, and faculty) who rated the same applicants in each circuit.

### MPI predictive validity

#### Preliminary validity evaluation

We examined the predictive validity of the 2013/2014 admitted cohort against performance in first year medical school courses. We evaluated predictive validity by regressing MPI total score against a five-station OSCE for first year medical students within the Arts in Science of Clinical Medicine year 1 course (ASCM year 1) and against year 1 in-programme didactic courses. The multiple linear regression model examined the prediction of MPI total score after controlling for undergraduate grade point average (GPA), medical college admission test (MCAT) sub-scores (Verbal Reasoning, Physical Sciences, and Biological Sciences), File Review total score, and Age. The OSCE consists of five examiners evaluating applicants across clinical scenarios intended to test physical examination skill and communication skills in the form of history-taking. While communication skills were scored across all stations, two stations examined history-taking specifically while the other stations focused on basic physical examination and rudimentary clinical judgment. Stations were scored using global ratings and we constructed three criterion measures: 1) average global rating score across all stations, 2) average global rating on the two history-taking stations, and 3) average global rating on the physical examination stations. We corrected correlations for range restriction [13] but not for reliability since the number of stations in the OSCE and MPI are fixed.

### Institutional recruitment and resourcing assessment outcomes associated with MPI implementation

To assess institutional impact of MPI implementation upon recruitment and resourcing, we assessed relevant outcomes across the pre-MPI implementation (2012/2013) and MPI implementation (2013/2014) admission cycles. Prior to MPI implementation, we used a traditional admission interview format. This format employed ‘open-file interviewing’ with two (faculty member and second year medical student) interviewers reviewing applicants’ admission files pre-interview for background information and generation of interview questions. Pre-interview file review is a common medical school practice [14]. The MPI innovation implemented in 2013/2014 is described in Appendix A and Fig. 1. We compared recruitment acceptance yields across these admission cycles. Admission recruitment acceptance yield was calculated; recruitment acceptance yield = total applicant admission acceptances/total applicant admission offers. We compared admitted medical student cohorts’ gender distribution, GPA and MCAT scores and interviewer cadres’ size and demographics across these admission cycles.

MPI implementation was deemed programme evaluation with results secondary to quality assurance activities and exempt from research ethics review. This determination was made by our local University of Toronto Health Sciences Research Ethics Board. ASCM year 1 OSCE validity evaluation received approval via the University of Toronto Research Ethics Board.

## Results

### MPI reliability results

We report variance due to factors that could contribute to error including: applicants, interview day, circuit, station, and item (Table 1). The variance component indicates the amount of error in measure due to each factor and interactions between the factors. For perfect reliability, all of the variance (i. e. 100 % of variation in scores) would be attributable to true differences between applicants. For implementation administration, MPI inter-interview reliability was 0.56, i. e. the average score across all four stations had a reliability of 0.56 indicating moderate reliability. The reliability of a single station was 0.12. The reliability of the Likert scale items within a single station was 0.92.

**Table 1:** Variance components for full implementation generalizability analysis

Facet	Raw	% Variance
Day (d)	0.000	0.000
Circuit:Day (c:d)	0.012	0.566
Applicant:Circuit:Day	0.360	17.094
Station	0.000	0.000
Item:Station	0.000	0.011
Day x Station	0.053	2.539
Day x Item	0.003	0.164
Circuit x Station:Day	*0.159*	*7.546*
Circuit x Item:Day	0.000	0.000
Applicant x Station:c:d	*1.089*	*51.738*
Applicant x Item:c:d	0.016	0.782
Station x Item	0.005	0.224
Day x Station x I	0.000	0.000
Circuit x Station x Item:d	0.027	1.290
Applicant x Station x Item:c:d	*0.380*	*18.047*

The majority of variance in scores was driven by the interaction between applicants x interview station (51 %) suggesting variation in scoring of applicants across different stations contributed most of the error in measurement. The omnibus interaction term (i. e. the variance due to random interaction between circuit, station, items, and applicants) was 18 % and true differences between applicants on its own was 17 %. The remainder of variance was attributed to small interactions with circuit and day of interview. The D‑study predicted eight stations to reach an inter-interview reliability of 0.7 or higher.

Item-total analyses showed all MPIs had similar item-total correlations (0.2–0.3). Similarly, the repeated measures ANOVA failed to detect a difference between station types in overall score (F(3,1680) = 0.811, *p* < 0.488). However, we also failed to detect a significant difference between medical student and resident raters compared with faculty when rating the same applicants (F(2243) = 0.126).

Correlations between total MPI score and admission file was 0.25 (*p* < 0.05) suggesting a moderate association; correction for restriction of range increased this to 0.34. The MPI self-reflection interview had marginally higher correlation (0.175) to the file review compared with other MPI interviews (0.15 to 0.16). The correlations with GPA and MCAT total score were not significant (r = −0.013, *p* < 0.25 for GPA and r = 0.10, *p* < 0.12).

### MPI predictive validity results

Total MPI scores had no significant correlations with grades on biomedical and basic science courses; correlations ranged between −0.1 and 0.1. However, MPI scores significantly predicted ASCM OSCE year 1 average global rating across all stations at 0.19 (*p* < 0.001), and average performance on history-taking stations at 0.20 (*p* < 0.001). Performance on physical examination stations was correlated at 0.16 (*p* < 0.05). While these correlations were low in absolute magnitude, correcting for range restriction showed the correlation with performance across all stations to be 0.30 and on history-taking stations at 0.33 with physical performance correlated at 0.24. Linear regression showed that the control covariate (GPA, MCAT sub-scores, age, file review total score) together had a R^2^ of 0.051 (i. e. predicted 5.1 % of variance in ASCM). Interestingly, t‑test of the coefficients showed that the pattern of prediction was driven by negative prediction of OSCE performance by age and performance on the Physical Sciences (i. e. Physics, Chemistry) sub-score of the MCAT. File review total score did not predict OSCE score. Addition of MPI total score increased the R^2^ of the whole model to 0.095, an R^2^ change of 0.043 which was significant (F(1248) = 11.86, *p* < 0.001). Comparison of the standardized coefficients showed the MPI total score to have the largest effect among the predictors. The complete regression model was significant (F(7247) = 3.69, *p* < 0.001). Coefficients and confidence intervals for each model are provided in Table 2 (MPI total score regressed against ASCM OSCE).

**Table 2:** Modified personal interview total score regressed against ASCM OSCE

Model		Unstandardized coefficients		Standardized coefficients	t	Sig	95.0 % confidence interval for B	
		B	SE	Beta			Lower bound	Upper bound
1	(Constant)	39.927	6.737	–	5.926	0.0001	26.658	53.196
	File review total	0.015	0.069	0.014	0.211	0.833	−0.122	0.151
	MCAT VR	0.147	0.142	0.069	1.035	0.302	−0.133	0.427
	MCAT PS	−0.360*	0.145	−0.194	−2.476	0.014	−0.646	−0.074
	MCAT BS	−0.096	0.149	−0.049	−0.642	0.521	−0.390	0.198
	Undergrad. GPA	−0.199	0.274	−0.047	−0.726	0.469	−0.739	0.341
	Age	−0.310**	0.110	−0.198	−2.826	0.005	−0.526	−0.094
2	(Constant)	33.799	6.833	–	4.947	0.0001	20.342	47.257
	File review total	0.007	0.068	0.007	0.108	0.914	−0.126	0.141
	MCAT VR	0.129	0.139	0.061	0.928	0.354	−0.145	0.403
	MCAT PS	−0.347*	0.142	−0.187	−2.436	0.016	−0.627	−0.066
	MCAT BS	−0.110	0.146	−0.056	−0.753	0.452	−0.398	0.178
	Undergrad. GPA	−0.221	0.268	−0.052	−0.822	0.412	−0.749	0.308
	Age	−0.326**	0.107	−0.208	−3.033	0.003	−0.538	−0.114
	MPI total	0.088**	0.026	0.208	3.432	0.001	0.037	0.138

*SE *standard error,* VR* verbal reasoning, *PS* physical sciences, *BS* biological sciences, *GPA* grade point average

### Institutional recruitment and resourcing results associated with MPI implementation

Applicant acceptance recruitment yield was maintained with MPI implementation, as were prestige markers reflected in admitted applicants’ GPA and MCAT scores. Rater resourcing for implementation of the new MPI format proved to be efficient with substantially fewer total interviewers (*n* = 130) required for assessment of slightly more (*n* = 13) applicants (Table 3).

**Table 3:** Impact of modified personal interview (MPI) on the MD admissions

Admissions cycle	2012/2013 (Pre-MPI)	2013/2014 (MPI)
# of applicants	3153	3463
% of male and female applicants	49:51	50:50
*Interview*		
# of interviewees	587	600
# of interviewers and demographics	290 145 faculty and 145 medical students (year 2)	160 87 faculty 36 residents and 37 medical students (year 4)
*First Year Class*		
# of offers, excluding deferrals to the following year	332	327
# of offers accepted	259	259
Acceptance yield	78 %	79 %
% of male and female accepts	49:51	50:50
Average accepted weighted grade point Average	3.92	3.94
Average MCAT Scores	Verbal reasoning – 7; Physical sciences – 7; and, Biological sciences – 9	Verbal reasoning – 10; Physical sciences – 11; and, Biological sciences – 12

## Discussion

The purpose of this study was to evaluate the combined admission issues of reliability, recruitment and resourcing for a modified personal interview that incorporated multiple-independent sampling while balancing resourcing and recruitment requirements. Additionally, we explored other psychometric properties of this admission interview format such as predictive validity.

Our MPI implementation measured reliability of 0.56 fell within the reliability range per number of interviews as predicted by Axelson and Kreiter [6] but is less than the traditionally accepted standards of 0.7 to 0.9 [13]. As noted with our D study, an MPI with eight interviews would attain a reliability of 0.7 or higher. An eight station MMI is not always feasible for medical schools and in some cases may not be desirable as schools may wish to spend the interview day hosting other recruitment activities for applicants. Furthermore, while high reliability is always a desirable goal, educational assessment has progressed beyond simply relying on measurement as the marker of assessment success [15]. The capability of educational assessments to incorporate institutional goals – in our case recruitment and resourcing – is important. For our context, the MPI implementation was associated with maintenance of our school’s acceptance yield and associated markers of high academic ability in our cohort such as MCAT and GPA scores. Just as important, it was a resource efficient method of interviewing students.

Furthermore, our study advances Axelson and Kreiter’s work [6] with our preliminary validity evidence of MPI score correlation with the ASCM year 1 OSCE. Prediction of performance is a significant marker of validity evidence since selection processes aim to choose the candidates most likely to succeed in medical school. The MPI shows early evidence of predicting aligned outcomes – communication skills performance – via an OSCE. Communication skills are best evaluated in face-to-face encounters and thus it is no surprise that MPI interview performance should predict performance in a similar in-programme simulated communication scenario. While the magnitude of the correlations is small, the score should be contextualized considering that the ASCM OSCE is a low stakes, short examination used for generating feedback and identifying skills deficits. Furthermore, the MPI was the significant positive predictor in the admission process. Like the MMI [16] it could be expected that the magnitude of MPI prediction could increase over time as more robust and reliable OSCE examinations are presented to our medical students. Validation is a continuing process that relies on the sufficiency of evidence to substantiate claims. Further work must clearly be done to build a chain evidence for the MPI’s validation.

Moreover, our MPI used caring and conscientiousness as the global competency creating an opportunity to help construct the importance of caring, compassion and conscientiousness as an institutional value for both faculty and learners. Thus, in addition to creating a more ‘human touch’ [2] for applicants, putting caring and conscientiousness as a focus of the MPI necessitated thoughtful consideration of the constructs by raters in every interview. We were able to leverage MPI rater training as a faculty, current medical student and resident educational development opportunity for sharing this instructional value through online and face-to-face sessions. These activities enriched the institutional value of the explicit attention afforded the institutional goals pertaining to recruitment and resourcing.

Our admission file assessment scores were not highly correlated with our MPI scores. This is likely a restriction of range issue but further evaluation of whether file assessment is a good screen for interview or if it in fact provides other information is worth investigating in the future. This finding presents our school with new admission resourcing and recruitment questions. Admission file review scores as a stand-alone method do not predict performance on the ASCM OSCE or indeed other assessments within our programme. However, ‘open-file interviewing’ via review of admission file materials within our self-reflection MPI station had similar measurement characteristics with other MPI stations. The resourcing and recruitment questions to now be addressed involve the heavy resource demand of the admission file review phase in the face of poor prediction capability. However, use of the applicant file in the interview has recruitment value as it is highly valued by our applicants. A next step is consideration of further use of ‘open-file interviewing’. For example, expansion of the MPI circuit to five interview stations to include a second MPI using ‘open-file interviewing’ warrants consideration.

Our focus upon institutional resourcing and recruitment factors ignored applicant resourcing and recruitment factors. Recruitment strategies integrated within interviews and interview day activities obviously requires applicants’ attendance and travel costs for many applicants. Attention to this matter is essential as medical education costs and medical student debt continues to rise. A next step will be in-depth qualitative investigation of the differential recruitment impact of the MPI and other recruitment strategies, including both institutional and applicant components. Investigation of recruitment strategies is essential as the field explores internet-based MMIs [17]. Internet-based interviewing may eliminate much of the need for applicant travel to attend campus-based admission interviews. To keep pace with technological innovation, comparative investigation of campus-based admission interviewing versus internet-based admission interviewing and their associated resourcing needs and recruitment strategies is necessary.

Our assessment of MPI implementation has limitations. We were interested in assessing the combination of admission issues of psychometric rigour (reliability and predictive validity), recruitment and resourcing with MPI implementation by comparing these issues and associated outcomes across pre-MPI and MPI admission cycles. We were able to do so with respect to recruitment and resourcing. However, we solely assessed reliability of administration of our implementation MPI and were unable to assess reliability of our pre-MPI use of a traditional admission interview format due to each applicant being interviewed by only a single interview station.

Our MPI seven-point Likert rating scale was selected to remain consistent with our large cadre of interviewers’ experiences in other local medical school in-programme assessment contexts. Selection of a broader Likert rating scale may have attained greater MPI reliability than the currently measured 0.56.

In addition, our resourcing measure related to the size of admission interviewer cadre alone, we did not include other resource components such as space and administrative staff requirements, and question development time as outlined by Rosenfeld et al. [18].

We believe this report provides evidence to advance uptake of Axelson and Kreiter’s ‘intermediate approach’ model for schools considering adoption of MIS-based interviewing in the face of questions regarding resourcing and/or recruitment [6]. Future study will explore psychometric factors such as longer-term MPI predictive validity. We will also investigate the differential value of various resourcing and recruitment strategies in preparation for a future without onsite medical school admission interviewing.

## Appendix A

### Modified Personal Interview (MPI) Innovation

We developed the Modified Personal Interview (MPI) for small-scale specialized medical student selection tasks (leadership [10] and MD PhD [12]). Furthering this model, we adapted the MPI to large-scale medical school admission interviewing.

### Interviewer cadre and training

This large-scale MPI adaptation required a large interviewer cadre to assess several hundred applicants for 259 medical school positions [19]. We included fourth-year medical students and residents as they could address applicant relevant issues and/or questions as a near-peer group [20]. MPI interviewer training was expanded to serve the training needs of this larger and more varied interviewer cadre. It included on-line (mandatory) and face-to-face components (optional). Interviewer training focused upon assessment rigour and recruitment. A generic online 24/7 available MPI training module oriented interviewers to the model, how to rate applicants, and how to conduct a warm, welcoming interview. In addition, on line modules for each of the four specific MPI interviews outlining specific competency assessment were offered. Face-to-face training sessions included simulated MPI scenarios performed live and videotaped for subsequent online upload and interviewer review. An MPI day orientation of approximately 20–30 min summarized important points. Online training was mandatory with 95 % of interviewers using this resource and 73 % of interviewers enrolled for face-to-face sessions.

### Competency range

Each MPI station focused on one specific competency, two common sub-competencies and a global competency for a total of four items evaluated per station. Specific competencies included: self-reflection, ethical-decision making, collaboration and values. Sub-competencies included maturity and communication/interpersonal skills with the global competency of caring and conscientiousness. Caring and conscientiousness were selected as the global competency subsequent to a Faculties of Health Sciences’ education retreat that recommended consideration of these constructs as common admission competencies across our university’s health sciences professional education faculties [21]. Applicants’ competencies were assessed via a 7‑point Likert rating scale. Each MPI had pre-determined interview questions and interviewers were trained to personalize the interaction by asking their own interview questions relevant to the specific MPI competency and applicant interview context.

One specific strategy employed to broaden the competency range assessed was ‘open-file interviewing’ by means of pre-interview file review by MPI interviewers. This approach enabled interviewers to sample a broad competency range personalized to applicants’ files, providing interviews with a ‘human touch’ [2]. However, it simultaneously introduced an interviewer bias via a halo bias caused by interviewers’ prior impressions acquired via pre-interview file review. For this MPI large-scale adaptation to maintain personalization but limit halo bias, we included ‘open-file interviewing’ with pre-MPI file review for only one of four MPI interviews. This interview rated self-reflection with applicants reflecting upon past activities noted in their files. This interview was examined carefully for differential functioning using item-total correlations, as it was unique within the MPI for utilizing pre-interview file review.

### MPI day organization

The MPI admission interview day comprised five simultaneous MPI circuits with each circuit consisting of four MPI stations; each MPI station is 12 min. For this large-scale MPI adaptation, we maintained the small-scale MPI four station circuit and interview duration [10, 12] for recruitment and resourcing reasons. As regards recruitment, this longer duration interview [22] would foster a ‘human touch’ [2] via increased opportunity for applicant/interviewer personal interactions and this small-scale circuit could be implemented alongside other MPI day recruitment campus activities. As regards resourcing, this small-scale circuit moderated rater resourcing demands. Each MPI circuit was repeated four times with 20 applicants per circuit and 80 applicants per day. An MPI day ran from 7:30am through to 1:30pm. Total applicant time for a MPI circuit was 54 min. Applicant movement through the MPI circuit was pre-determined with first year medical student hall monitors available to guide applicants through their circuit. Applicants’ MPI circuit flow was guided by a voice over recording including a welcome message and description of MPI timing. This MPI day organization enabled integration of the MPI with other recruitment activities including campus tours. Further, to enhance recruitment, an MPI circuit comprised an interviewer cadre of one fourth year medical student, one postgraduate medical trainee (resident) and two faculty members.
